# Epidemiological trends in abdominal trauma in Norway (2015–2023): a national register-based cohort study of severity, population-adjusted incidence, and short-term outcomes

**DOI:** 10.1016/j.lanepe.2026.101654

**Published:** 2026-03-25

**Authors:** Johannes Wiik Larsen, Kjetil Søreide, Jon Arne Søreide, Ingvild Dalen, Kenneth Thorsen

**Affiliations:** a Department of Gastrointestinal Surgery, Stavanger University Hospital, Stavanger, Norway; b Section for Traumatology, Surgical Clinic, Stavanger University Hospital, Stavanger, Norway; c Department of Clinical Medicine, University of Bergen, Bergen, Norway; d Section of Biostatistics, Department of Research, Stavanger University Hospital, Stavanger, Norway; e Stavanger Trauma Investigation Group (STING), Stavanger University Hospital, Stavanger, Norway

**Keywords:** Trauma, Abdominal, Epidemiology, Incidence, Mortality

## Abstract

**Background:**

Published data describing epidemiological trends of abdominal injury on a national level are scarce. This study aims to analyse trends in demographics, severity, population-adjusted incidences, and short-term outcomes of abdominal trauma based on national trauma register data.

**Methods:**

Observational, complete national cohort study of all consecutive traumatic incidents resulting in abdominal injuries reported to the National Trauma Register (NTR) of Norway between 2015 and 2023. The NTR has demonstrated an overall coverage rate of 92.2% of trauma patients, with excellent data accuracy. Standardised incidence rates were estimated using the direct method with standard populations. Temporal trends were evaluated in regression models.

**Findings:**

Abdominal injuries occurred in 9.0% (7086/78,416) of all registered traumatic incidents. Children represented 18.1% (n = 1275) of the patients, and 14.8% (n = 1047) were elderly. The median age was 35 years (interquartile range, IQR: 19–55) and increased by 0.4 years annually (95% confidence interval, CI: 0.0–0.8). Men accounted for 69.5% (n = 4926) and 30.5% (n = 2160) were female. Blunt injury type dominated with 85.3% (n = 6044). Traffic-related accidents were the most common mechanism (48.0%, n = 3401), followed by falls (26.9%, n = 1903). The median Injury Severity Score (ISS) was 10 (IQR 2–18), and 36.3% (n = 2567) displayed ISS>15 indicating severe injury. Polytrauma occurred in 20.6% (n = 1457) of trauma cases suffering abdominal injury. Age- and sex-adjusted incidence of all abdominal injuries was 14.9/100,000/year (95% CI: 14.5–15.3). The annual increase was 0.43 per 100,000 person-years (95% CI: 0.14–0.71) among children and 0.68 per 100,000 person-years (95% CI: 0.39–0.97) for elderly patients. The subgroup of solid organ injuries showed an adjusted incidence of 7.2/100,000/year (95% CI: 7.0–7.5), and hollow viscus injuries of 1.2/100,000/year (95% CI: 1.1–1.3). Overall 30-day mortality was 3.4% (n = 240), with no statistically significant change during the study period.

**Interpretation:**

Trauma patients presenting with abdominal injury are getting older. One third sustained severe injuries and one in five patients suffered polytraumatic injuries. The incidence of abdominal trauma increased over the last decade, predominantly amongst elderly patients and children. The mortality was low, and stable over time.

**Funding:**

None.

**Research in context**

**Evidence before this study**

We searched PubMed for nationwide, population-based studies on abdominal injuries from 1 January 2000 to 4 March 2026, using the search terms “abdominal injury”, “trauma”, “epidemiology”, and “incidence” without language restrictions. Previous articles consist of single-centre series, retrospective multi-centre collaborations, or aggregated register data from trauma networks on singular or selected organ injuries only or with focus on management strategies. We found no national studies on population-based age- and sex-adjusted incidence rates covering all abdominal injury. Comprehensive, population-based analysis on trends in epidemiology and mortality across age-groups for patients with abdominal injuries are lacking.

**Added value of this study**

This population-based, complete cohort study on prospective registered data from a national trauma register in a uniform health care system presents novel data on adjusted incidence rates of abdominal trauma admitted to hospital. Time trends over the last decade describing the epidemiology of abdominal injury, including the subgroups of solid-organ injury and hollow viscus injury are presented for children, adults, and elderly patients. This contemporary analysis, derived from a nationwide dataset, supports planning and capacity estimations throughout the trauma chain of survival and enables benchmarking of outcomes across geographical regions.

**Implications of all the available evidence**

The data shows increasing incidences of abdominal injury over time, particularly in elderly patients but also in children. An overall low proportion of severely injured patients and the stable low mortality rate over time must not obscure the fact that certain subgroups, such as elderly patients injured in low-energy falls and those with severe penetrating trauma, more often experience fatal outcomes and continue to pose significant challenges for response capacity and resource management within the trauma healthcare system. Focus on prevention and effective in-hospital management are vital to optimise outcomes for patients with abdominal injuries.

## Introduction

Globally, injuries still represent one of the leading causes of premature deaths, life-years lost, and disability in the young, even though injury-related age-standardised Disability-Adjusted Life Year (DALY) rates seem to have been decreasing over the last two decades.^1^ Major demographic shifts during this period have impelled changes in trauma epidemiology, with trauma becoming the leading cause of death in people up to 50 years of age in certain regions.^2^

Planning trauma systems and emergency care starts with understanding the epidemiology of trauma and the burden to the system.^3^ Given the continuously changing global security landscape, the trauma system should be equipped to address not only injuries caused by traffic accidents and falls, including low-energy mechanisms in more fragile patients in both ends of the age-spectrum, but also the consequences of large-scale international armed conflicts and unforeseen terrorist incidents^4^ producing mass casualties from both blunt and penetrating injury mechanisms.^5^, ^6^, ^7^ Hence, there is a need to investigate and understand the epidemiology of trauma within any regional population.

Abdominal injuries represent approximately 6–7% of all traumatic injuries in a Northern European setting,^8^, ^9^, ^10^ but abdominal organ injuries can pose a time-critical challenge in trauma management with risk of death.^11^^,^^12^ The broad spectrum of possible organ injuries in this body region, many of which can lead to circulatory collapse, mandates a trauma system prepared for rapid assessment and timely interventions to prevent morbidity and mortality.

This study provides a contemporary and comprehensive epidemiological overview of injured patients admitted to hospital with abdominal trauma in a universal health care system with a designated trauma system covering the diverse geography, climate, and population-density variation across Norway. The study aims to analyse trends in demographics, severity, population-adjusted incidences, and short-term outcomes of abdominal trauma based on national trauma register data.

## Methods

### Study design and period

This population-based, nationwide observational cohort study is a retrospective analysis of prospectively collected data reported to the NTR between 1 January 2015 and 31 December 2023.

The Strengthening the Reporting of Observational Studies in Epidemiology (STROBE) guidelines^13^ for cohort studies and the REporting of studies Conducted using Observational Routinely collected health Data (RECORD) statement^14^ were adhered to.

### Setting

Norway covers 385,207 square kilometres with a mixed rural and urban settlement of 5,165,802 inhabitants in 2015, increasing to 5,550,203 inhabitants in 2023, for a 7.4% increase over the period.^15^ The universal, publicly funded healthcare system of Norway encompasses four healthcare regions (Northern, Central, Western, and Southeast) and five university hospitals as designated trauma centres. Additionally, a total of 35 acute care hospitals of variable size and catchment areas are admitting trauma and emergencies, and provide initial trauma care with subsequent transfer to a trauma centre if necessary. Due to the mergers or closures of smaller hospitals, this number was reduced to 33 from 2017. No other parallel healthcare services that manage trauma patients exist in Norway. The National Trauma Plan was implemented in 2007 and updated in 2016,^16^ to ensure identical criteria for Trauma Team Activation (TTA) across all hospitals receiving trauma patients, and uniformity in the offered services of trauma care. The NTR began full-scale registration of trauma patients on 1 January 2015,^17^ recording data from all trauma receiving hospitals nationwide.^18^

Inclusion criteria are TTA at the index hospital prompted by the prespecified national criteria.^17^ Additionally, injured patients not received by a trauma team despite meeting the criteria, or treated for injuries specified in additional inclusion criteria will be searched for by local Abbreviated Injury Score (AIS) code-certified registrars. The additional inclusion criteria are: All patients with New Injury Severity Score (NISS) > 12, all penetrating injuries to the head, neck, torso, and extremities proximal to the elbow or knee, and all patients with a head injury with AIS severity code ≥3. As the NTR aims to register all pre-hospital deaths in which pre-hospital resources were mobilised, this has constituted an inclusion criterion for the NTR since 2015.

All available information, including patient records, imaging studies, operative notes, and autopsy data, is investigated by local registrars who follow the patient until end of the hospital stay, and 30 days post-injury to assess for 30-day mortality. Institutional trauma register databases are prospectively maintained and report to the national register on an annual basis. The NTR has demonstrated a 100% coverage rate for patients received via TTA, and an overall coverage rate of 92.2% when including patients beyond those received by the trauma team.^19^ A validation study comparing the register data with electronic patient records found excellent data accuracy.^20^

### Participants and data description

All patients with documented abdominal injury of any severity according to the AIS 2005 revision, update 2008^21^ were identified from the national trauma register database. Each trauma case is assigned a unique ID number in the NTR, allowing assessment of the full treatment pathway across different hospitals. Furthermore, a patient-specific ID number can be used to identify repeated traumas for the same patients. Data from all hospital stays related to each trauma case were sorted chronologically and merged, ensuring no duplicate cases were generated in instances where patients were transferred between hospitals. The first admission to a trauma centre is considered the index hospital stay. In lieu of a trauma centre admission, the first hospital will be considered the index hospital stay. Patient characteristics and pre-hospital information were obtained from the first admission. Trauma severity and vital parameters were obtained from the index admission. Outcome measures including Length Of Stay (LOS), Intensive Care Unit (ICU) days, ventilator days, and 30-day mortality combined information from all hospital stays.

### Variables and definitions

The AIS scoring system grades each specific organ injury from anatomical location (body regions) in severity ranging from minor (grade 1) to maximum (grade 6) and in case of insufficient information to assign a true AIS severity, coded 9. AIS ≥3 identifies serious, severe, critical, and unsurvivable injuries.^21^ NTR includes up to 25 AIS scores per trauma.

Solid organ injury was defined as injury to one or more of the solid organs in the abdominal cavity: liver, spleen, kidney, pancreas, mesentery, omentum, or adrenal glands. Hollow viscus injury was defined as injury to one or more of the hollow viscera in the abdominal cavity: stomach, duodenum, small bowel, colon, rectum, biliary tract, bladder, or ureter. Abdominal vessel injury was defined as an injury to identified vascular structures in the abdominal cavity not categorised under other specified organ injuries. Ano-genital injuries included injury to the anus, perineum, or reproductive organs, both internal and external. Diaphragmatic injuries are classified as thoracic injuries according to the AIS and are not included in this material.

The date of each injury was based on the injury start time registered, and the cases were classified according to the calendar year of the injury dates. Age was measured in years at the time of injury. Age groups were defined as children (≤16 years of age), adults (17–64 years), and elderly (≥65 years of age).

Pre-injury comorbidity was assessed using the American Society of Anesthesiologists (ASA)^22^^,^^23^ physical status classification score, describing the patient's overall physical health before injury ranging from 1 (healthy patient) to 5 (moribund patient), indicated in patient records at index hospital.

The dominant injury type was defined by the highest AIS score, with penetrating trauma considered dominant when blunt and penetrating injuries have identical scores. Low-energy falls were those from the same level or up to one metre. Falls from higher than one metre or combined with speed were classified as high-energy.

Vital parameters, i.e. systolic blood pressure (mmHg), respiration rate given in breaths per minute (bpm), and Glasgow Coma Scale (GCS)^24^ for assessing consciousness on a scale from 3 up to 15, were obtained on admission at first hospital. GCS ≤8 serves as a marker of severe cognitive impairment.

To assess overall trauma severity, the Injury Severity Score (ISS)^25^ was used. Summing the squares of the highest AIS scores in the three most severely injured body regions provides a score ranging from 0 to 75, where ISS >15 indicates a severely injured patient, >25 indicates critically injured patient and 75 is unrevivable. If any AIS severity score is 9, ISS is undefined. The New Injury Severity Score (NISS),^26^ a later modification of the ISS, calculating the sum of the squares of the three most severe injuries, regardless of the body region in which they occur, was also used. This also range from 0 to 75, and the score of NISS >12 chosen as inclusion criteria by the NTR indicates moderate to severe injury. Polytrauma was defined as an injury with an AIS score >2 in two or more body regions.^27^

Probability of survival was assessed using the Trauma and Injury Severity Score (TRISS),^28^ as a variable given in the dataset provided by the NTR. The TRISS formula for survival prediction (also referred to as Probability of Survival; P_S_) combine patient's age, ISS, and type of injury (blunt/penetrating trauma), and the admission physiology as scored by the Revised Trauma Score (RTS).^29^ The RTS is the categorical expression of GCS, systolic blood pressure, and respiratory rate. The TRISS score gives an estimate ranging from 0 (no probability of survival; P_S_ 0.0) to 1 (100% probability of survival; P_S_ 1.0).

Mortality was defined as any death occurring within the first 30 days after the trauma event (date of injury). Patients registered as dead on arrival were described as such according to the Utstein template for uniform reporting.^30^ Foreign citizens who returned to their country of origin within 30 days following injury and remained alive at the time of departure were censored as survivors at date of departure.

### Statistical analysis

Categorical data were presented as numbers and percentages, and continuous data as medians and interquartile ranges (IQR), or mean with standard deviation (SD). Comparisons between blunt and penetrating injuries in demographic, clinical, and trauma management variables were performed with Chi-square tests for proportions, Kruskal–Wallis tests for medians and independent samples t-test with Welch correction for mean values. Proportions of categories for injury mechanism and intent of the trauma were compared between age groups (children, adults, and elderly) using Chi-square tests.

Binary and multinomial logistic regression and quantile (median) regression were applied to evaluate temporal changes in the distribution of selected characteristics, i.e., patient sex, age, ASA score, injury mechanism, injury type, ISS, and polytrauma, within the trauma population. Predicted proportions/medians were estimated for each calendar year in models where the selected characteristic entered as the dependent variable, and calendar year entered as a categorical independent variable, and plotted with 95% confidence intervals (CI) based on cluster-robust standard errors allowing for repeated traumas for the same patients.

Crude incidence rates were calculated per 100,000 person-years using annual national population data from Statistics Norway,^15^ based on January 1st counts for each year (2015–2024) and converted to mid-year estimates.

Age- and sex-standardised incidence rates were calculated using the direct method, based on observed counts and standard populations structured in combinations of sex and five-year age bands (0–4 years, 5–9 years, and so on), and presented with exact 95% CI. The World Health Organization (WHO) World Standard Population (2000–2025),^31^ the European Standard Population 2013 (ESP 2013),^32^ and the United States (US) 2000 Standard Population^33^^,^^34^ were used to enable global comparisons. Age-adjusted incidence rates were estimated separately for males and females using the same approach. Patients with missing data on age (n = 29) were excluded from these calculations.

Time trends in age-adjusted incidence rates of total abdominal traumas and the subgroups of solid organ injuries and hollow viscus injuries were analysed using Poisson regression modelling based on aggregated data (in combinations of age groups, sex, and calendar year), with mid-year population count as the exposure variable. Age adjustment was performed by including age in five-year bands as a categorical predictor in a fully saturated model. In this way we mimicked the direct standardisation method above. The highest age-group was ≥90 as in the ESP 2013 standard population. For hollow viscus injuries we had to combine the lowest age categories (<15 years) due to low numbers and inestimable standard errors. Adjusted marginal incidence rates were estimated for each sex and for the total sample and plotted against calendar year with 95% CI obtained by the delta method and by weighting with the population counts. For age adjustment in the total sample, the counts and populations were combined for males and females.

Annual incidence rates for the three age groups (children, adults, and elderly) were estimated using Poisson regression with age group, calendar year (categorical) and their interaction as independent variables and with the mid-year population of these age groups as exposure.

Overall annual mortality rates (in percent) were estimated using binary logistic regression, with calendar year as a categorical predictor and applying cluster-robust standard errors allowing for repeated traumas per patient. Crude mortality rates and rates adjusted for patient and trauma characteristics (patient sex, age, ASA score, injury mechanism, injury type, ISS, and polytrauma) were presented as marginal predicted proportions with 95% CI. Mortality within subgroups defined by the same characteristics was assessed in separate univariable logistic regression models. Overall comparisons between subgroups were reported as odds ratios with 95% CI. Within-subgroup temporal patterns were examined in models including calendar year (categorical), the subgroup variable, and their interaction as independent variables, from which marginal predicted proportions over time were plotted with 95% CI.

Linear temporal trends were assessed by modelling year as a continuous predictor to estimate the average annual change. All analyses were performed with all available cases, and the numbers of missing values are indicated for all results. p-values <0.050 were considered statistically significant.

The SPSS® version 29 for Mac (IBM, Armonk, New York, USA) was used for descriptive statistics and simple tests. R version 4.5.0^35^ with the package epitools was used to assess standardised incidence rates. Stata version 18.0^36^ was used for regression analysis and figures.

### Ethics approval

The Regional Committee for Medical and Health Research Ethics Western Norway approved this study (REK # 185,928). Upon inclusion, all patients in the Norwegian National Trauma Register (NTR) receive written information about the register, enabling access to registered data with the possibility of anonymisation. All data extracted for this study were de-identified.

### Role of the funding source

No funding.

## Results

A total of 7086 trauma events involving abdominal injuries were identified, representing 6936 unique injured patients. Patients who experienced one traumatic incident with abdominal injury accounted for 6885 (99.3%) cases, while 51 patients (0.7%) had multiple admissions, ranging from two to 21 separate traumatic incidents. Abdominal injuries occurred in 9.0% (7086/78,416) of all registered traumatic incidents. The frequency of abdominal injuries in age categories is shown in the study flow-chart (Supplementary Figure S1), and baseline characteristics are presented in Table 1.

**Table 1:** Characteristics of 7086 traumatic abdominal injuries in 6936 unique patients registered in the National Trauma Registry of Norway during 2015–2023, with comparison between dominating injury types.

	Total	Blunt	Penetrating	p-value
Trauma cases, n (%)^a^	7086 (100)	6044 (85.3)	981 (13.8)	
Patient characteristics				
Male, n (%)	4926 (69.5)	4179 (69.1)	698 (71.2)	0.205
Age in years, median (IQR)	35 (19–55)	36 (18–56)	34 (24–47)	0.390
*Missing data, n (%)*	29 (0.4)	20 (0.3)	6 (0.6)	
Age category, n (%)				<0.001
*Children*	1275 (18.1)	1189 (19.7)	71 (7.3)	
*Adult*	4735 (67.1)	3869 (64.2)	830 (85.2)	
*Elderly*	1047 (14.8)	966 (16.0)	73 (7.5)	
ASA score, n (%)				<0.001
*1*	4551 (65.4)	4053 (67.8)	498 (51.2)	
*2*	1799 (25.9)	1454 (24.3)	343 (35.3)	
*3*	579 (8.3)	449 (7.5)	130 (13.4)	
*4*	28 (0.4)	26 (0.4)	2 (0.2)	
*Missing data, n (%)*	129 (1.8)	62 (1.0)	8 (0.8)	
Trauma management, clinical characteristics and trauma severity				
Transport time in minutes, median (IQR)				
*EMCC alerted to arrival ED*	58 (37–91)	62 (40–95)	39 (26–62)	<0.001
*Departure scene to arrival ED*	22 (11–48)	24 (12–49)	14 (7–26)	<0.001
*Missing data, n (%)*	1761 (24.8)	1484 (24.6)	224 (22.8)	
Trauma team activation, n (%)	6570 (93.4)	5615 (93.3)	920 (94.1)	0.521
*Missing data, n (%)*	51 (0.7)	27 (0.4)	3 (0.3)	
SBP (mmHG), median (IQR)	130 (115–144)	130 (115–145)	130 (116–142)	0.845
*SBP < 90, n (%)*	310 (4.4)	261 (4.4)	46 (4.8)	0.574
*Missing data, n (%)*	197 (2.8)	147 (2.4)	29 (3.0)	
RR (bpm), median (IQR)	20 (16–24)	20 (16–24)	20 (16–24)	0.432
*RR < 10 or ≥ 30, n (%)*	855 (13.1)	731 (13.1)	123 (14.1)	0.416
*Missing data, n (%)*	580 (8.2)	445 (7.4)	106 (10.8)	
GCS, median (IQR)	15 (15–15)	15 (15–15)	15 (15–15)	0.286
*GCS < 9, n (%)*	320 (4.5)	279 (4.7)	39 (4.0)	0.366
*Missing data, n (%)*	118 (1.7)	83 (1.4)	12 (1.2)	
Single abdominal injury, n (%)	2934 (41.4)	2673 (44.2)	228 (23.2)	<0.001
ISS, median (IQR)	10 (2–18)	12 (5–20)	4 (1–9)	<0.001
*ISS > 15, n (%)*	2567 (36.3)	2401 (39.8)	140 (14.3)	<0.001
*Missing data, n (%)*	16 (0.2)	13 (0.2)	2 (0.2)	
NISS, median (IQR)	12 (3–22)	14 (5–22)	4 (1–12)	<0.001
*NISS > 12, n (%)*	3371 (48.1)	3132 (51.9)	239 (24.4)	<0.001
*Missing data, n (%)*	16 (0.2)	13 (0.2)	2 (0.2)	
Polytrauma, n (%)	1457 (20.6)	1351 (22.4)	91 (9.3)	<0.001
TRISS, median (IQR)	0.99 (0.98–1.00)	0.99 (0.97–1.00)	0.99 (0.99–1.00)	<0.001
TRISS, mean (SD)	0.96 (0.12)	0.96 (0.12)	0.97 (0.12)	<0.001**^∗^**
*Missing data, n (%)*	196 (2.8)	125 (2.1)	10 (1.0)	
Outcomes				
LOS in days, median (IQR)	4 (2–8)	5 (2–9)	3 (2–6)	<0.001
*Missing data, n (%)*	32 (0.5)	14 (0.2)	3 (0.3)	
Ever ICU, n (%)	4480 (72.4)	3859 (73.2)	599 (68.4)	0.003
*Days in ICU, median (IQR)*	2 (1–4)	2 (1–4)	2 (1–3)	<0.001
*Missing data, n (%)*	897 (12.7)	775 (12.8)	105 (10.7)	
Ever ventilator, n (%)	1098 (15.7)	863 (14.4)	230 (23.9)	<0.001
Days on *ventilator, median (IQR)*	3 (2–10)	4 (2–12)	2 (1–3)	<0.001
*Missing data, n (%)*	94 (1.3)	58 (1.0)	20 (2.0)	
30-day mortality				
*Overall, n (%)*	240 (3.4)	203 (3.4)	36 (3.7)	0.630
*DOA at scene, n (%)*	10 (0.1)	7 (0.1)	2 (0.2)	0.489
*DOA at ED, n (%)*	27 (0.4)	19 (0.3)	8 (0.8)	0.120
*Missing data, n (%)*	113 (1.6)	85 (1.4)	11 (1.1)	

Data presented as count (percentage) for categorical variables and as median (interquartile range) for continuous variables, unless otherwise specified. Percentages estimated from non-missing cases, except percentages of missing cases based on total cases. Comparisons between dominating injury type (blunt and penetrating) performed with Pearson Chi square test (categorical variables) and Mann–Whitney U tests (continuous variables) except ∗independent samples t-test with Welch correction.

Abbreviations: AIS, Abbreviated Injury Scale; ASA, American Society of Anesthesiologists physical status classification system; bpm, breaths per minute; DOA, dead on arrival; ED, emergency department; EMCC, Emergency medical call centre; GCS, Glasgow Coma Scale; ICU, intensive care unit; ISS, Injury Severity Score; IQR, interquartile range; LOS, length of hospital stay; n, number of trauma cases; NISS, New Injury Severity Score; RR, respiration rate; SBP, systolic blood pressure; TRISS, Trauma and Injury Severity Score; SD, standard deviation.

a Dominating injury type (blunt/penetrating) missing information in 61/7086 (0.9%) of cases.

**Fig. 1:**
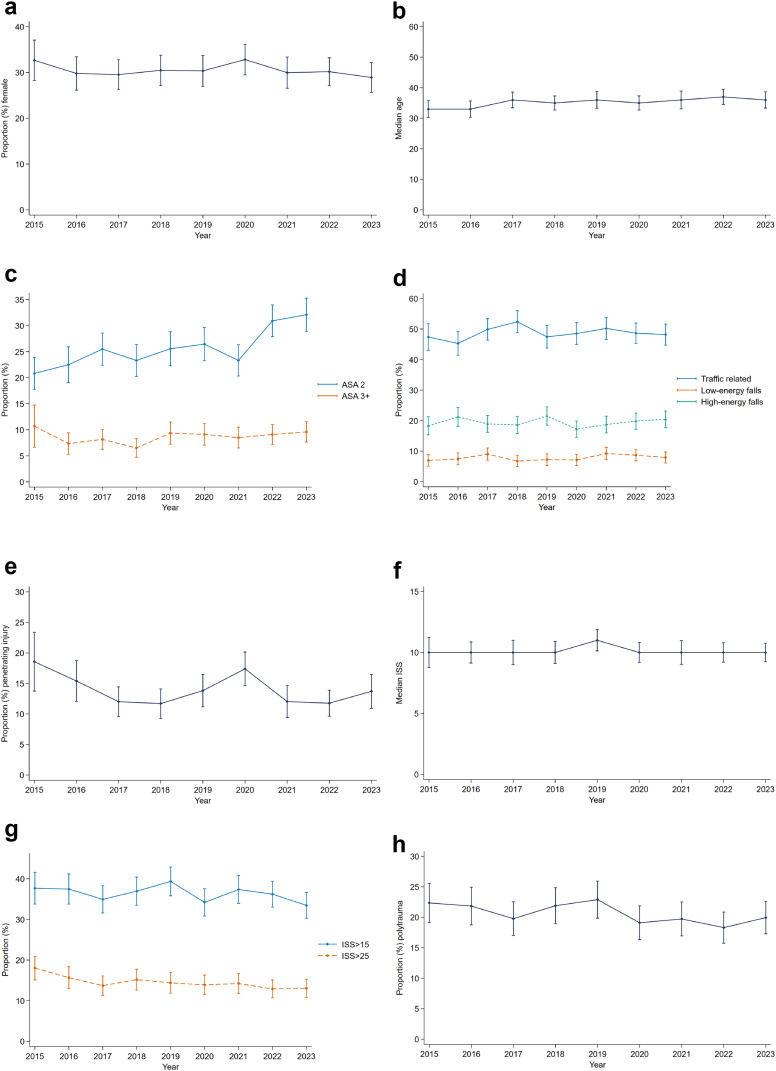
**Time trends in patients with abdominal injury**. All eight panels display year-by-year aggregated data for the given characteristic in patients with abdominal injuries during the study period. Error bars (solid lines with caps) represent the 95% confidence interval. Panel a: Proportion of female patients. Panel b: Median age, given in years. Panel c: Proportion of ASA score 2 and 3+. Panel d: Proportion of injury mechanism. Low-energy falls were those from the same level or up to one metre. High-energy falls were those from higher than one metre or combined with speed. Panel e: Proportion of penetrating injury. Panel f: Median Injury Severity Score (ISS). Panel g: Proportion of severe and critical injury. ISS >15 indicates a severely injured patient. ISS >25 indicates critically injured patient. Panel h: Proportion of polytrauma.

Changes over time in selected characteristics are displayed in Fig. 1. The proportion of females versus males was stable over time. The median age increased by 0.4 years annually (95% CI: 0.0–0.8) during the study period, (p = 0.036). A decreasing proportion of polytrauma was noted (p = 0.046).

Solid organ injuries were found in 48.9% (3463/7086) of the cases. Hollow viscus injuries were observed in 8.8% (621/7086), and combined solid organ and hollow viscus injuries were found in 4.2% (297/7086) of the cases. Solid organ injury was more frequent in children (53.4%, 681/1275) and elderly patients (50.5%, 529/1047) than in adults (47.3%, 2241/4735) (p < 0.001). Hollow viscus injury displayed a different pattern, being more frequent in adults (9.8%, 466/4735) and elderly patients (8.9%, 93/1047) than in children (4.6%, 59/1275) (p < 0.001).

### Associated injuries

Associated injury in other body regions than the abdominal cavity was found in 74.1% (5252/7086). The frequency of associated injuries differed across age categories, ranging from 59.1% (753/1275) in children up to 86.7 (908/1047) in elderly patients (p ≤ 0.001). Thoracic injuries were sustained in 46.0% (3260/7086) of the trauma cases, lower extremities injuries in 31.0% (2198/7086), upper extremities 27.8% (1970/7086), spine 20.1% (1421/7086), head 19.2% (1357/7086), face 17.1% (1215/7086), and neck injuries in 2.7% (193/7086).

### Mechanism

Blunt trauma was the predominant injury type, representing 85.3% (6044/7086) of all trauma events. Injury mechanisms are displayed in Table 2. The annual proportions of traffic-related injuries, high-energy falls, and low-energy falls are displayed in Fig. 1, with no statistically significant change throughout the study period. Low-energy falls displayed a significantly higher frequency in female (8.9%, 193/2160) versus male patients (7.2%, 355/4926) (p = 0.012).

**Table 2:** Mechanisms and intents of 7086 traumatic abdominal injuries in 6936 unique patients registered in the National Trauma Registry of Norway during 2015–2023, with comparison across age categories.

	Total (n = 7086)^a^	Children (n = 1275)	Adult (n = 4735)	Elderly (n = 1047)	p-value
Injury mechanism					
Traffic related accident^b^	3401 (48.7)	673 (53.7)	2267 (48.5)	449 (43.4)	<0.001
*Motor vehicle*	1655 (23.7)	153 (12.2)	1203 (25.8)	294 (28.4)	
*Motorcycle*	630 (9.0)	127 (10.1)	460 (9.9)	41 (4.0)	
*Bicycle*	733 (10.5)	294 (23.5)	377 (8.1)	59 (5.7)	
*Other*	187 (2.7)	51 (4.1)	121 (2.6)	14 (1.4)	
*Pedestrian hit by motorised vehicle*	196 (2.8)	48 (3.8)	106 (2.3)	41 (4.0)	
Fall	1903 (27.2)	364 (29.1)	1076 (23.0)	456 (44.1)	<0.001
*Low-energy*	548 (7.8)	84 (6.7)	228 (4.9)	235 (22.7)	
*High-energy*	1355 (19.4)	280 (22.3)	848 (18.2)	221 (21.4)	
Impact–blunt object	574 (8.2)	134 (10.7)	396 (8.5)	41 (4.0)	<0.001
Stab wound	841 (12.0)	37 (3.0)	738 (15.8)	59 (5.7)	<0.001
Gunshot wound	55 (0.8)	2 (0.2)	49 (1.0)	4 (0.4)	0.002
Explosion	13 (0.2)	0 (0.0)	12 (0.3)	1 (0.1)	0.134
Other	199 (2.8)	43 (3.4)	132 (2.8)	24 (2.3)	
Missing data	100 (1.4)	22 (1.7)	65 (1.4)	13 (1.2)	
Intention					
Accidental	5859 (84.4)	1217 (97.0)	3668 (79.2)	952 (92.2)	<0.001
Self-harm	573 (8.2)	12 (1.0)	498 (10.8)	62 (6.0)	<0.001
Violence	493 (7.1)	24 (1.9)	448 (9.7)	16 (1.6)	<0.001
Other	21 (0.3)	1 (0.1)	17 (0.4)	2 (0.2)	
Missing data	140 (2.0)	21 (1.6)	104 (2.2)	15 (1.4)	

All data presented as counts (percentages). Percentages estimated from non-missing cases, except percentages of missing cases based on total cases. Comparison between age groups performed with Pearson Chi Square test. Abbreviations: n, number of trauma cases.

a Patient age missing in 29/7086 (0.4%) of cases.

b Driver or passenger of indicated vehicle.

Falls showed higher severity with a median ISS of 14 (IQR 5–20), compared to traffic-related injury (median ISS of 10; IQR 2–21) and all other mechanisms (median ISS of 5; IQR 1–16) (p < 0.001).

Violent intent was encountered more frequently in males (8.6%, 422/4926) compared to females (3.3%, 71/2160) (p < 0.001). Intention of self-harm showed the opposite pattern being more frequent in females (12.2%, 264/2160) as compared to males (6.3%, 309/4926) (p < 0.001).

### Penetrating injury

Penetrating injury was the dominating injury type in 13.8% (981/7086) of the trauma cases. The number of unique patients with penetrating injury was 848, with 34 patients (4.0%) having multiple, separate incidents with penetrating injury to the abdomen, ranging from two to 21 incidents. The proportion of penetrating injury was stable during the study period (Fig. 1).

Stab wounds mainly affected adults, who showed higher pre-injury ASA scores than patients with blunt injuries (Table 1). Almost half of the penetrating injuries were caused by the intention of self-harm, ranging from 5.8% (4/69) amongst children, up to 60.3% (44/73) in the elderly, surpassing violence as a cause of abdominal injury in patients older than 16 years of age. Violence caused abdominal injury in 36.3% (356/981) of the penetrating injuries.

### Severity

The abdominal cavity showed the highest, or tied for highest, severity score according to the AIS in 67.4% (4776/7086) of the cases. The proportion of abdominal injuries presenting with AIS ≥3 in this cohort was 34.8% (2467/7086). Table 3 shows the proportion of the different organ injuries presenting with AIS ≥3.

**Table 3:** Frequencies, proportions, and crude incidence rates of specific abdominal organ injuries among 7086 abdominal traumas in 6936 unique patients registered in the National Trauma Registry of Norway during 2015–2023.

Abdominal organ systems	Number of injuries	Injuries with AIS ≥3^a^, n (%)	Proportion among abdominal injuries (N = 7086) (%)	Proportion among total injuries in registry (N = 72,774) (%)	Crude incidence rate per 100,000 person-years
Solid organs	3463	1991 (57.5)	48.9	4.8	7.2
*Spleen*	1531	913 (59.6)	21.6	2.1	3.2
*Liver*	1279	603 (47.1)	18.0	1.8	2.7
*Kidney*	918	576 (62.7)	13.0	1.3	1.9
*Mesentery*	232	61 (26.3)	3.3	0.3	0.48
*Adrenal gland*	188	10 (5.3)	2.7	0.3	0.39
*Pancreas*	91	34 (37.4)	1.3	0.1	0.19
*Omentum*	83	13 (15.7)	1.1	0.1	0.17
Hollow viscus	621	338 (54.4)	8.8	0.9	1.3
*Small bowel*	253	175 (69.1)	3.6	0.3	0.53
*Colon*	230	92 (40.0)	3.2	0.3	0.48
*Stomach*	96	43 (44.8)	1.4	0.1	0.20
*Duodenum*	76	24 (31.6)	1.1	0.1	0.16
*Bladder*	60	34 (56.7)	0.8	0.08	0.12
*Rectum*	22	10 (45.5)	0.3	0.03	0.05
*Ureter*	19	11 (57.9)	0.3	0.03	0.04
*Biliary tract*	19	5 (26.3)	0.3	0.03	0.04
*Appendix*	2	0 (0.0)	0.03	0.003	0.004
Abdominal wall	3270	35 (1.1)	46.1	4.5	6.8
Retroperitoneal haematoma	148	0 (0)	2.1	0.2	0.31
Abdominal vessels^b^	287	278 (96.9)	4.1	0.4	0.60
Ano-genital^c^ including urethra	181	16 (8.8)	2.6	0.2	0.38

Abbreviations: AIS, Abbreviated Injury Scale; n, number of trauma cases.

a AIS ≥3 indicates serious, severe, or critical injuries.

b Named vessels not included in other organ injury description.

c Injuries to the anus, perineum, or male/female reproductive organs.

The median ISS of 10 was stable during the study period (Fig. 1). The proportion of patients with ISS over 15 was 36%, showing a decreasing trend over the study period, although this was not statistically significant. The annual proportion of patients with ISS over 25 decreased significantly (p = 0.005). The blunt injury type showed a higher proportion of severely injured patients compared to the penetrating injury type. In patients with penetrating injury, only 5.4% (53/981) had an ISS over 25. Of note, this was significantly higher with 15.9% (963/6044) in blunt injuries (p < 0.001).

Children suffered a lower trauma burden with 26.5% (338/1275) of cases with ISS over 15, compared to 36.3% (1721/4735) and 47.6% (498/1047) in adults and elderly patients, respectively. In patients with ISS over 25, children accounted for 6.0% (77/1275), which was lower than in adults at 16.1% (761/4735) and the elderly at 17.4% (182/1047) (p < 0.001).

### Incidences

Crude incidence of trauma cases resulting in any abdominal injury was found to be 14.5 per 100,000 person-years (95% CI: 14.1–14.8). Solid organ injuries displayed a crude incidence of 7.2 per 100,000 person-years (95% CI: 6.9–7.4), and hollow viscus injuries a crude incidence of 1.3 per 100,000 person-years (95% CI: 1.2–1.4). Age- and sex-adjusted incidences for abdominal injuries adjusted towards standard populations for international comparisons are displayed in Table 4.

**Table 4:** Standardised incidence rates of abdominal injuries and subgroups based on data from the Norwegian National Trauma Registry during 2015–2023.

Standard populations	Abdominal injuries			Solid organ injuries			Hollow viscus injuries		
	Total	Male	Female	Total	Male	Female	Total	Male	Female
WHO World	14.9 (14.5–15.3)	20.0 (19.4–20.6)	9.8 (9.3–10.3)	7.2 (7.0–7.5)	10.3 (9.9–10.8)	4.1 (3.8–4.4)	1.2 (1.1–1.3)	1.7 (1.5–1.9)	0.7 (0.6–0.8)
ESP 2013	14.4 (14.0–14.7)	20.1 (19.5–20.7)	8.6 (8.3–9.0)	7.1 (6.9–7.3)	10.2 (9.8–10.6)	4.0 (3.7–4.2)	1.3 (1.2–1.4)	1.8 (1.6–2.0)	0.8 (0.7–0.9)
US 2000	14.6 (14.3–15.0)	20.4 (19.8–21.0)	9.1 (8.7–9.5)	7.2 (6.9–7.4)	10.4 (10.0–10.8)	4.1 (3.8–4.3)	1.3 (1.2–1.4)	1.8 (1.6–2.0)	0.8 (0.6–0.9)

Standardised incidence rates given as number of injuries per 100,000 person-years and presented with 95% confidence intervals. Rates were standardised to the WHO World Standard (2000–2025), the European Standard Population (ESP 2013), and the US 2000 Standard Population using direct standardisation, giving age- and sex-standardised total rates and age-standardised rates for each sex.

**Fig. 2:**
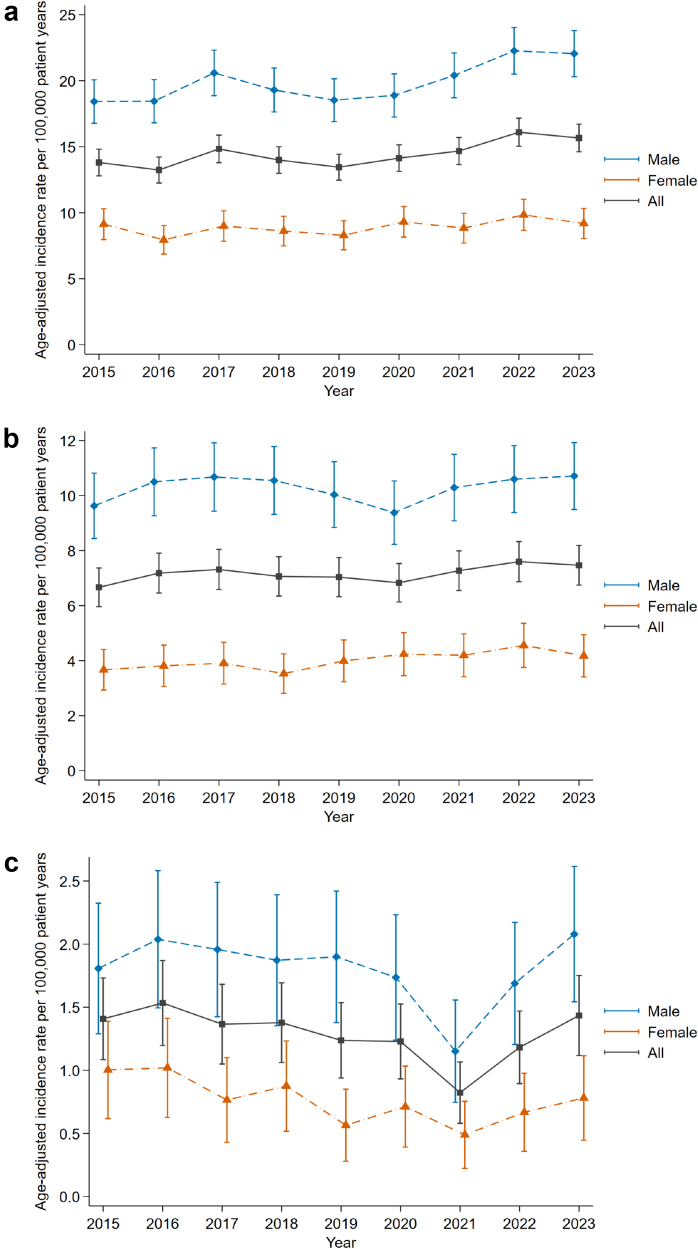
**Annual age-adjusted incidence rates of abdominal injuries per 100,000 patient years**. Error bars (solid lines with caps) represent the 95% confidence interval. Panel a: All abdominal injuries, overall and stratified by sex. Panel b: Solid organ injuries, overall and stratified by sex. Panel c: Hollow viscus injuries, overall and stratified by sex.

The annual age-adjusted incidence rates of all abdominal injuries, solid organ injuries, and hollow viscus injuries, are depicted in Fig. 2. The increasing incidence over time for all abdominal injuries was statistically significant (p < 0.001), with an annual increase of 0.27 per 100,000 person-years (95% CI: 0.14–0.40). The increase was observed mainly amongst male patients with an increase of 0.43 per 100,000 person-years per year (95% CI: 0.21–0.65) (p < 0.001). Female patients showed an estimated, but not statistically significant, increase of 0.11 per 100,000 person-years per year (95% CI: −0.04 to 0.26) (p = 0.151). In solid organ injury, no statistically significant increase was found in male patients (p = 0.546), nor in female patients (p = 0.063). Hollow viscus injury showed a stable incidence during the study period in both sexes.

**Fig. 3:**
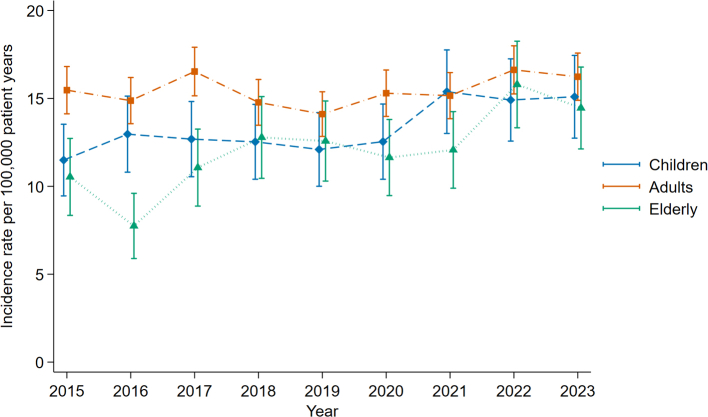
**Annual incidence rates of abdominal injuries per 100,000 patient years in three age categories**. Annual incidence rates for all abdominal injuries in children (≤16 years of age), adults (17–64 years of age), and elderly patients (≥65 years of age). Error bars (solid lines with caps) represent the 95% confidence interval.

The observed incidence rate of all abdominal injuries in age categories is presented in Fig. 3. While the incidence was stable over time in adults, an annual increase of 0.43 per 100,000 person-years per year (95% CI: 0.14–0.71) was observed in children (p = 0.003). For elderly patients, the increase amounted to 0.68 per 100,000 person-years per year (95% CI: 0.39–0.97) annually (p < 0.001).

### Mortality

**Fig. 4:**
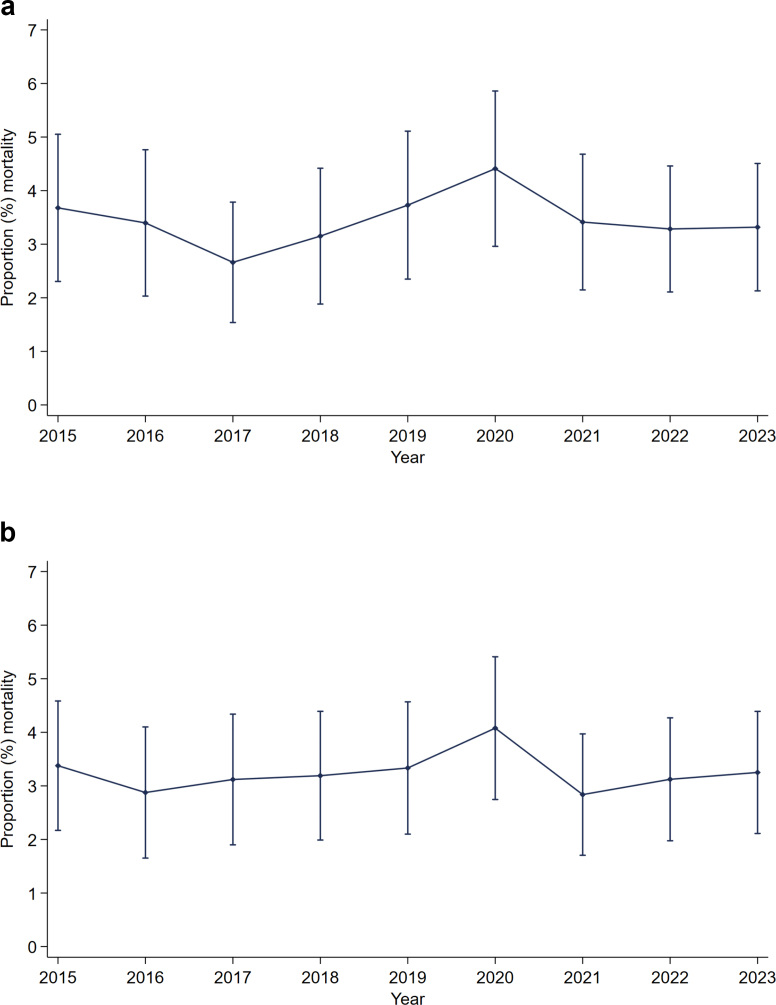
**Annual mortality rates in patients with abdominal injuries**. Error bars (solid lines with caps) represent the 95% confidence interval. Panel a: Unadjusted mortality rate, given in percent. Panel b: Adjusted mortality rate, given in present. Adjusted for sex, age, American Society of Anesthesiologists (ASA) score, Injury Severity Score (ISS), polytrauma, dominant injury type, and injury mechanism.

The overall 30-day mortality was 3.4% (95% CI: 3.0–3.9) representing 240 fatalities among 6973 trauma cases with known outcome. The abdominal cavity was the body region with the highest AIS score in 38.3% (92/240) of the deceased. Mortality rates remained stable during the study period (Fig. 4A). (p for linear trend = 0.861). Adjusting for patient characteristics (sex, age, ASA, ISS, polytrauma, dominant injury type, and injury mechanism) did not change this picture (p = 0.937 for linear trend; see Fig. 4B and Supplementary Table S1). Time trends for the mortality rate in subgroups of patients with abdominal injury are presented in Supplementary Figure S2.

In univariable analyses, higher mortality risks were associated with higher age, higher ASA and ISS scores, and with sustaining polytrauma and low-energy falls; see Supplementary Table S1. In particular, traumas with ISS >25 had 28 times higher odds of 30-day mortality than those with ISS ≤15 (OR 28.1; 95% CI: 18.7–42.4; p < 0.001). Comparing all with ISS >15 versus ISS ≤15, we found 14 times higher odds of death (OR 14.0; 95% CI: 9.4–20.8, p < 0.001). Median ISS of the deceased was 34 (IQR 21–50). No difference in mortality rates was found between the sexes (p = 0.973). Median age of the deceased patients was 54 years (IQR 34–79). Mortality rates ranged from 0.6% (8/1262) in children to 2.9% (136/4649) in adults and 9.2% (95/1037) in elderly patients.

No statistically significant difference in mortality rate was found between penetrating (3.7%, 36/970) and blunt (3.4%, 204/6003) injury type, but gunshot wounds stood out with a mortality rate of 10.9% (6/55). Mortality rate after low-energy falls (6.6%, 36/544), was almost double compared to high-energy falls (3.5%, 46/1323). Of the 36 fatalities following low-energy falls, 31 occurred in elderly patients and five in adults, with a median age of 81.5 (IQR 73–88), a median ISS of 16.5 (IQR 9–26), and 27.8% (10/36) suffering polytrauma. The abdominal region had the highest AIS in 58.3% (21/36) of these patients.

## Discussion

This study found an increasing incidence of abdominal injuries over the last decade, with a stable mortality rate over time. The frequency of abdominal injuries among all traumatic incidents of 9.0% is higher than previously reported from regional efforts in Norway,^8^ while data from Scotland found abdominal injuries at a frequency of 7.2%.^10^ The inclusion criteria of the Norwegian trauma register may partly explain the higher frequency of abdominal injuries found in this complete cohort, and likely also more accurately depict the actual workload that this group poses in hospitalised trauma patients.

Solid organ injuries are far more frequent than hollow viscus injuries. The latter is not always identified using Computed tomography (CT) with sensitivity being lower for diagnosing bowel injuries.^37^^,^^38^ It is therefore noteworthy that this often occult injury was twice as prevalent in adults and elderly patients as in children, a consideration of clear relevance to clinical practice. Although the incidence of abdominal injuries increased, the proportion of polytraumatised patients and patients with an ISS >25 decreased. One possible explanation for this is the ongoing transition to a newer and safer car fleet in our society. A longstanding focus on safety systems and policies, together with continuous changes in infrastructure, has led to significant reductions in road traffic deaths over time in high-income countries,^39^ which we believe are mirrored in the findings on abdominal injury. Even though treatment is not addressed in this study, a decrease in the most severe injuries would typically reduce both the rate of emergency procedures and the exposure to polytraumatised patients for the individual surgeons.

Blunt injury type more often led to abdominal injury in the more vulnerable age groups of children and elderly patients than penetrating injury. Blunt injury caused a higher frequency of multiple abdominal injuries and polytrauma with higher severity (higher median ISS), resulting in more use of ICU resources and more extended hospital stays. Despite these differences, patients with penetrating injuries had approximately half the transport time of those with blunt injuries. With penetrating injury only accounting for 13.8% of the injuries in this series, this can be a case of “fear of the unfamiliar”, with these injuries being highly respected in every level of the trauma chain of survival. Rightfully so, one might argue, when looking at the high mortality in critically injured patients (ISS >25) after penetrating injury type. Still, this warrants the question of whether more can be done after blunt injury, especially when looking at differences in outcome with a focus on the elderly after low-energy falls.

The adjusted incidence of abdominal injuries is increasing in Norway, with an annual increase of 0.39 per 100,000 person-years over the study period. The increase was found in children and with an even bigger increase among elderly patients. The maturation of the NTR, including the effort to more systematically identify and include undertriaged patients across all trauma receiving hospitals,^17^ might explain some of the increase but this effect should not differ between age groups. The rising incidence in children could be due to some increased availability and improved quality of diagnostics (e.g. ultrasound, CT, and Magnetic Resonance Imaging (MRI) scans) potentially lowering the threshold for cross-sectional imaging use in children leading to a higher detection rate of injuries,^40^ but during our study period these minor technical advancements can only explain a small fraction of the increase. We have no data suggesting an increased risk behaviour in Norwegian children in this period. On the contrary, there is a growing concern of higher screen-time and less physical activity in children today. One could speculate that increasing focus on adhering to treatment guidelines and documentation requirements over time have lowered the threshold for referring these patients to hospital care, hence, more of the low-grade injuries are encompassed by the NTR. The amount and proportion of elderly patients exposed to major trauma are increasing rapidly,^41^ and the increased risk of severe injury in geriatric patients has previously been shown in national data from Norway.^42^ This study shows that this increase is evident for abdominal injury as well. The explanation for this pattern may lie in an ageing population accumulating a higher degree of comorbidities, with low-energy falls increasing as trauma mechanism. Logically, high-energy falls should inflict more injury, but when the ageing population with reduced physiological reserves are exposed to even low-energy injury, the outcome may be fatal. This in contrast to the young and healthy individual who may suffer a much lower trauma burden despite exposure to the same amount of energy.

The estimates of adjusted incidence for all abdominal injuries and subgroups enable comparisons between geographical regions. Adjusting towards different standard populations further simplifies such comparisons. Factors that are paramount in obtaining data to facilitate this include a well-functioning trauma system with modern diagnostic capabilities, coupled with a trauma register that ensures good coverage across the population to capture data on the complete national cohort. In a global context, the combination of these factors is rare and may partially explain why similar numbers are not available from other complete national datasets.

It is worth noting that the incidence calculations in this study reflect a society characterised by favourable socioeconomic conditions, restrictive traffic legislation with a zero-tolerance policy for drugs and alcohol, a modern car fleet, and low rates of violent events combined with strict firearms regulations. From a patient and healthcare perspective, this is clearly a ‘less is more’ scenario, where zero injuries would be preferable. On the other hand, from a trauma system perspective, a low incidence, even though increasing, means challenging conditions for educating and preparing the system for optimal management of these injuries and ensuring favourable outcomes for each patient. In particular, the low rate of penetrating injuries stands out compared to other regions in the world.^43^

Overall mortality in this cohort was low, reported at 3.4%. Among all included patients, the majority suffered a relatively low injury severity, with only 36.3% having an ISS >15. The mortality in the severely injured group (ISS >15) was 8.2%, and might be more representative when comparing the outcome with other national data.^10^ Even though the overall median injury severity and mortality are low, these factors remain high within certain subgroups, as exemplified by a mortality rate of almost double in the low-energy fall group compared to high-energy falls. This most likely reflect the reduced physiological capacity of elderly patients, more pre-existing conditions, frailty and polypharmacy. Continued focus on preventive measures, including avoiding falls among the elderly, and improving road traffic safety, is essential to decrease the incidence of abdominal injuries across Norway, while simultaneously maintaining preparedness for proper management of abdominal injuries.^44^

In addition to the findings discussed above, the strengths of this study deserve emphasis. Pertinent data were collected prospectively in a nationwide network, comprising the entire population as a part of the universal, publicly funded health care system in Norway. Data completeness is generally very high (92.2–100%),^17^ and missing data are encountered only for a few variables.

Despite these strengths, some limitations warrant consideration. The inclusion criteria in the NTR can pose a limitation in itself when attempting to calculate incidences, highlight the workload such injuries represent for the trauma system, and present the outcome of abdominal injuries. Isolated single abdominal injuries from a suspected low-energy mechanism, can pose a clinical challenge despite not being included in the NTR if not met with a trauma team at admission. Examples of such injuries may consist of rare, single solid organ injuries in children after ordinary activities that do not reach the threshold of NISS > 12.^45^ Calculations on national data may therefore be less suitable for capturing every abdominal injury than analyses based on local datasets. The maturation of the trauma system reporting to the NTR possibly leading to better adherence to the criteria for trauma team activation, the continuing focus on identification and inclusion of undertriaged patient, and increased effort to include prehospital deaths^17^ might serve as a limitation when analysing time trends in altering both the nominator and denominator in calculations on incidence-rates, severity (including polytrauma-rates) and mortality.

In conclusion, Norwegian trauma patients suffering abdominal injury are presenting with increasingly higher age over time. One third suffered severe injuries (ISS >15). The proportions of critical injuries (ISS >25) and polytraumatic injuries seem to decline. The incidence of abdominal trauma has increased over the last decade, with an evident rise in elderly patients in particular and amongst children. Overall mortality was low and remained stable over time, with no difference between blunt and penetrating injury types.

## Contributors

All authors contributed to the conception and design of the study.

JWL, and KT performed the data collection.

JWL, ID and KT have directly accessed and verified the underlying data.

JWL, ID performed data management and analysis.

All authors contributed to the interpretation of data.

JWL draughted the first manuscript, and all authors contributed to the revision of both textual and conceptual content. All authors were responsible for the decision to submit the manuscript.

## Data sharing statement

The data utilised in this study are available from the national trauma register (NTR) of Norway trough application, following approval from the appropriate ethics committee. The data are thereby not publicly available without project specific approval.

## Declaration of interests

All authors have completed the ICMJE disclosure form and declare: KS was regular board member of the inaugural steering committee from 2013 to 2017 in the Norwegian National Trauma Register (NTR). KT was alternate board member from 2020 to 2025, and is currently a regular board member of the NTR steering committee. All steering committee roles were unpaid.

The NTR steering committee had no role in the research question, data analyses, interpretation, nor in the decision to publish. The Authors bear the sole responsibility for the data analyses and interpretation as presented.
