# Supplementary material for: Epidemiological trends in abdominal trauma in Norway (2015–2023): a national register-based cohort study of severity, population-adjusted incidence, and short-term outcomes
Source: Lancet Reg Health Eur. 2026 Mar 25;65:101654. doi: 10.1016/j.lanepe.2026.101654 (PMC13053684; doi:10.1016/j.lanepe.2026.101654)
Supplement: Supplementary Material [file mmc1.pdf]

Supplementary material

Table of content

Table S1…………………………………………………………………........2
Figure S1…………………………………………………………………...4
Figure S2……………………………………………………………..…….5

Table S1. Factors associated with the risk of 30-day mortality. Results from univariable logistic regression analyses for 6793[1)] trauma cases (within 6826 patients) from the National Trauma Registry in Norway, 2015-2023.

Factor	N	Number who died	Proportion (%) with (95% CI)	OR (95% CI)	P-value
Sex					
Male	4845	167	3·4 (3·0–4·0)	1 (ref)	
Female	2128	73	3·4 (2·7–4·3)	1·00 (0·75–1·32)	0·973
Age group					< 0·001
Children	1262	8	0·6 (0·3–1·3)	0·21 (0·10–0·43)	< 0·001
Adults	4649	136	2·9 (2·5–3·5)	1 (ref)	
Elderly	1037	95	9·2 (7·5–11·1)	3·35 (2·55–4·39)	< 0·001
ASA					< 0·001
1	4490	96	2·1 (1·8–2·6)	1 (ref)	
2	1778	69	3·9 (3·1–4·9)	1·85 (1·35–2·53)	< 0·001
3+	602	63	10·5 (8·2–13·3)	5·35 (3·83–7·48)	< 0·001
ISS					< 0·001
ISS ≤ 15	4427	28	0·6 (0·4–0·9)	1 (ref)	
ISS > 15 and ≤ 25	1524	54	3·5 (2·7–4·6)	5·77 (3·64–9·15)	< 0·001
ISS > 25	1007	153	15·2 (13·1–17·6)	28·1 (18·7–42·4)	< 0·001
Polytrauma					
No	5541	85	1·5 (1·2–1·9)	1 (ref)	
Yes	1432	155	10·8 (9·3–12·5)	7·79 (5·94–10·22)	< 0·001
Dominant injury type					
Blunt	6003	204	3·4 (3·0–3·9)	1 (ref)	
Penetrating	970	36	3·7 (2·7–5·1)	1·10 (0·76–1·58)	0·627
Injury mechanism					< 0·001
Traffic-related	3360	97	2·9 (2·4–3·5)	1 (ref)	
Low-energy falls	544	36	6·6 (4·8–9·0)	2·38 (1·61–3·53)	< 0·001

High-energy falls	1323	46	3·5 (2·6–4·6)	1·21 (0·85–1·73)	0·291
Other/unknown	1663	58	3·5 (2·7–4·5)	1·22 (0·87–1·70)	0·251

1) Total possible N in these analyses was 6973, due to mortality status being unknown for 113 (1·6%) cases. Estimated proportions and odds ratios (OR) presented with 95% confidence intervals (CI) based on cluster robust standard errors allowing for correlation between repeated traumas for the same patients.

Fig. S1. Study flow chart.

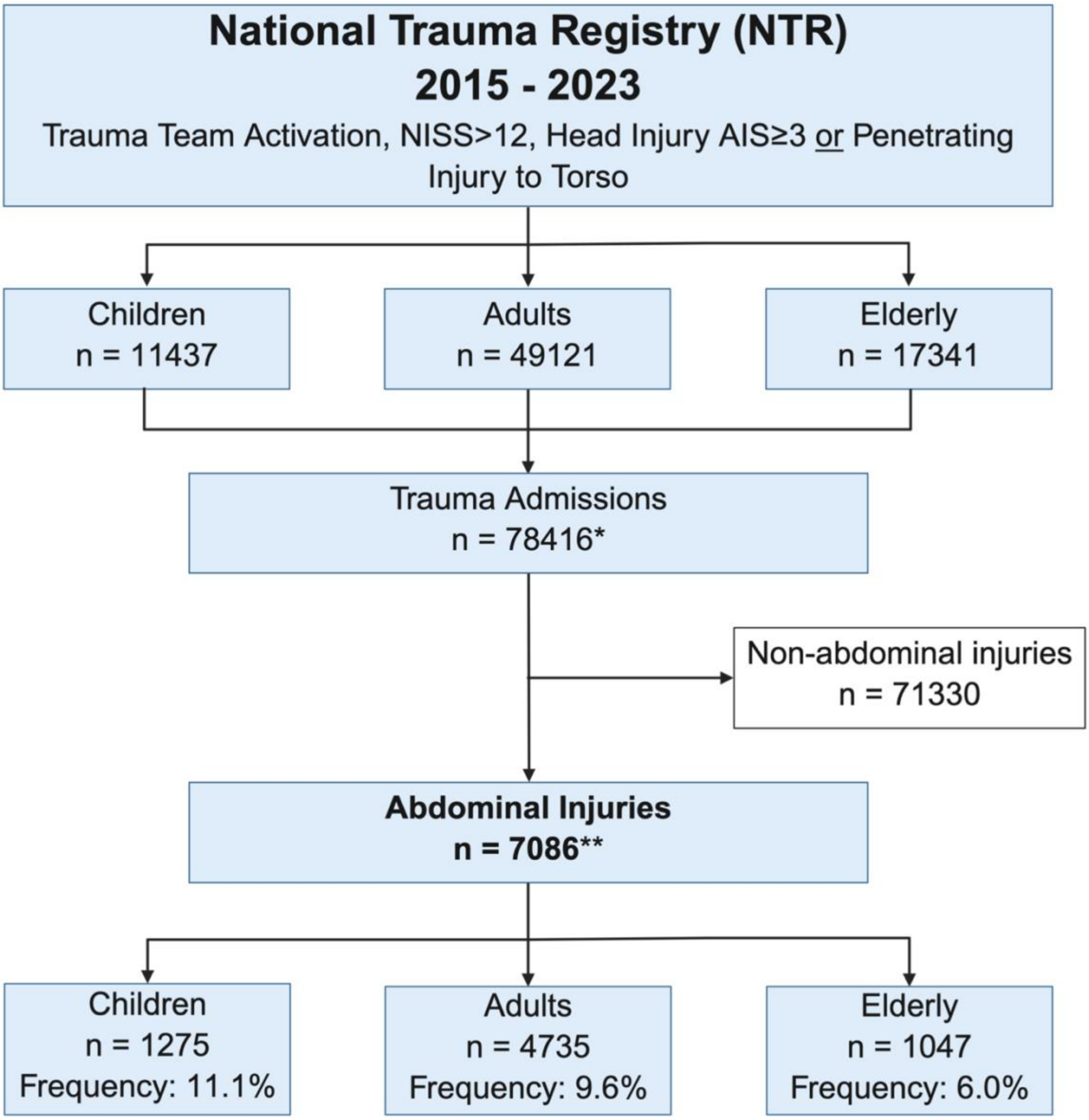

Patient selection and the frequencies of abdominal injuries are shown for children, adults, and elderly patients. Inclusion criteria for National Trauma Registry (NTR) are listed beneath the heading. Frequency = Frequency of abdominal injuries amongst all cases with traumatic injury in each age category over the study period.

*Patients with missing data on age (n = 517), are not displayed under age categories.

**Patients with missing data on age (n = 29), are not displayed under age categories.

Fig. S2. Time trends of mortality rate in patients with abdominal injury.

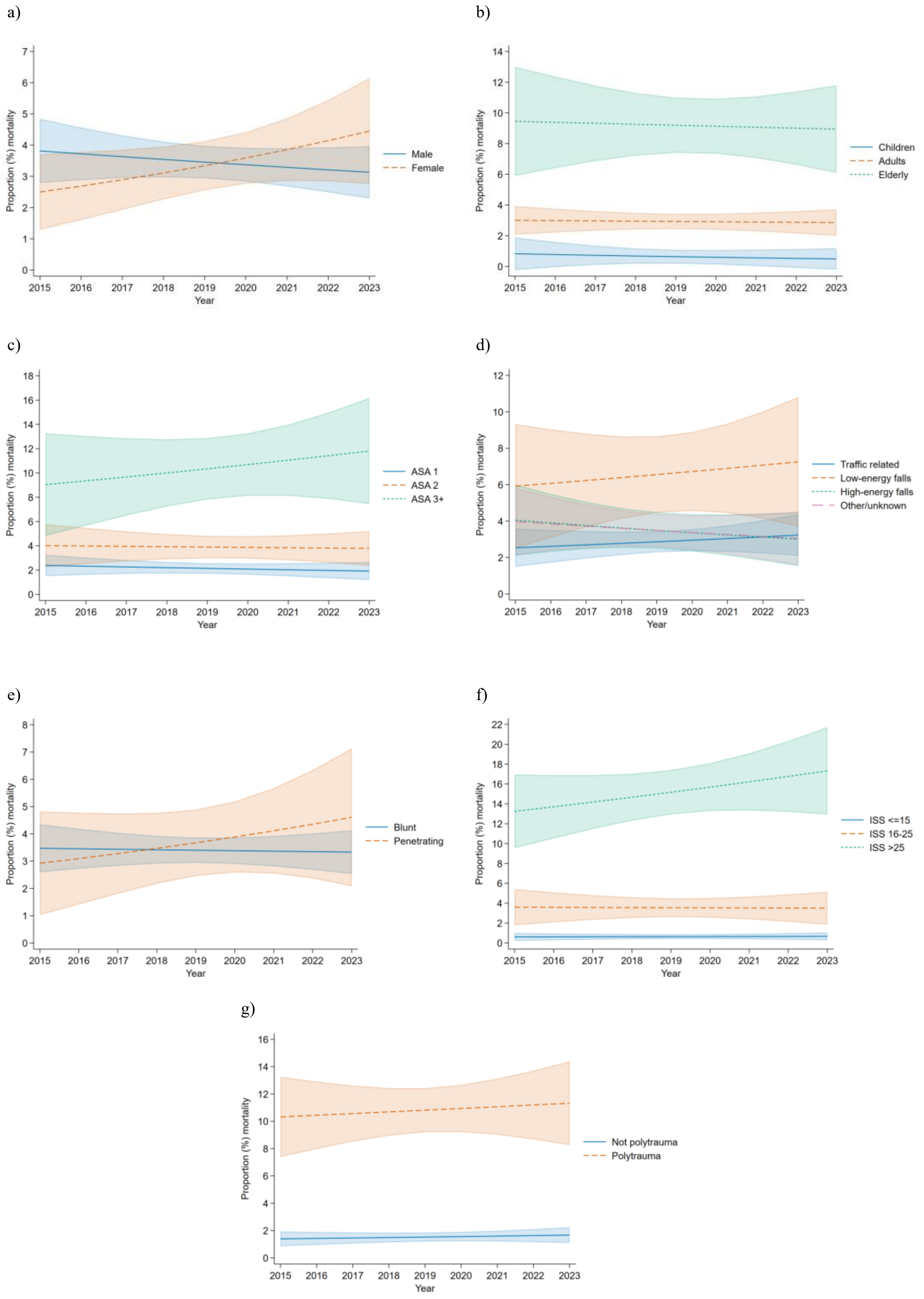

All seven panels display year-by-year mortality rate (percent) for the given characteristic in patients with abdominal injuries during the study period. Shaded area represents the 95% confidence interval.

Panel A: Mortality rate of male and female patients.
Panel B: Mortality rate in the age categories children (≤ 16 years of age), adults (17–64 years of age), and elderly patients (≥ 65 years of age).
Panel C: Mortality for American Society of Anesthesiologists (ASA) score 1, 2, and 3+.
Panel D: Mortality rate for injury mechanisms traffic injuries, low-energy falls, high-energy falls, and other. Low-energy falls were those from the same level or up to one meter. High-energy falls were those from higher than one meter or combined with speed.
Panel E: Mortality rate for dominant injury type, blunt and penetrating.
Panel F: Mortality rate for categories of Injury Severity Score (ISS), 0-15, 16-24, 25+.
Panel G: Mortality rate for polytrauma and not polytrauma.
